# Supplementary material for: Stable topical application of antimicrobials using plumbing rings in an ex vivo porcine corneal infection model
Source: PLoS One. 2025 Apr 3;20(4):e0319911. doi: 10.1371/journal.pone.0319911 (PMC11968109; doi:10.1371/journal.pone.0319911)
Supplement: S1 Protocol — (PDF) [file pone.0319911.s001.pdf]

Feb 02, 2025

An Optimized Porcine Corneal *Ex Vivo* Model Using Plumbing Rings and Medical Adhesive for Controlled Localized Infection with Topical Treatments

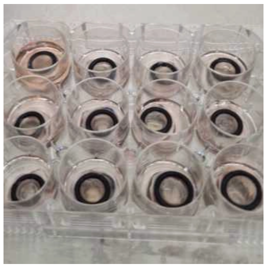

DOI
dx.doi.org/10.17504/protocols.io.eq2ly6nkmgx9/v1

Daniel Foulkes[1], Keri Mclean[1], Tarunima Sharma[1], David Fernig[1], Stephen Kaye[1]

[1]University of Liverpool

Daniel Foulkes
University of Liverpool

OPEN ACCESS

DOI: dx.doi.org/10.17504/protocols.io.eq2ly6nkmgx9/v1

Protocol Citation: Daniel Foulkes, Keri Mclean, Tarunima Sharma, David Fernig, Stephen Kaye 2025. An Optimized Porcine Corneal Ex Vivo Model Using Plumbing Rings and Medical Adhesive for Controlled Localized Infection with Topical Treatments. **protocols.io** https://dx.doi.org/10.17504/protocols.io.eq2ly6nkmgx9/v1

License: This is an open access protocol distributed under the terms of the **Creative Commons Attribution License**, which permits unrestricted use, distribution, and reproduction in any medium, provided the original author and source are credited

Protocol status: Working
We use this protocol and it's working

Created: December 02, 2024

Last Modified: February 02, 2025

Protocol Integer ID: 113423

Keywords: Ex vivo, infection model, cornea, antimicrobial, porcine, Microbial keratitis, Host-pathogen interactions, Pseudomonas aeruginosa, Localized pathogen inoculation, Plumbing ring model, Antimicrobial treatment assessment, Corneal opacity

Funders Acknowledgements:
Fight for Sight
Grant ID: 5175 / 5176

Abstract

Infection models are essential tools for understanding host-pathogen interactions and developing novel therapies. Ex vivo infection models offer significant ethical and practical advantages, enabling more accurate and physiologically relevant insights compared to traditional cell culture systems.

The versatility of porcine corneal ex vivo models has made them valuable for studying a range of pathogens, including *Pseudomonas aeruginosa*, *Staphylococcus aureus*, *Streptococcus pneumoniae*, *Candida albicans*, *Acanthamoeba*, and viruses such as Herpes Simplex and adenovirus. Here, we present an optimized *ex vivo* protocol that addresses several limitations, using plumbing rings and medical adhesive to enhance experimental accuracy and reproducibility.

The application of a 10 mm plumbing ring to the centre of the cornea allows localized inoculation, preventing the "run-off" of topically added aqueous solutions and ensuring that pathogens remain confined to the site of infection. Additionally, this setup enables precise maintenance of therapeutic agents on the corneal surface, allowing for more reliable assessments of antimicrobial effects.

For analysis, we describe methods for colony-forming unit (CFU) quantification within the cornea, evaluation of corneal opacity, and histological analysis using hematoxylin and eosin staining to assess infection progression over 48 hours.

This improved, accessible, and cost-effective *ex vivo* model provides a standardized platform for evaluating potential treatments, ultimately supporting clinical translation and reducing the burden of microbial keratitis.

Materials

Required Materials

- Whole pig eyes (obtainable from local abattoirs).
- Surgical scissors, forceps and scalpel (size 10) (Fisher scientific).
- Phosphate-buffered saline (PBS), 10% (v/v) iodinated povidone (Ecolab) and ethanol.
- 10 mm Plumbsure Rubber O rings (from Hardware stores such as B&Q).
- LIQUIBAND 0.8 g Exceed topical skin adhesive (Advanced Medical Solutions).
- Luria–Bertani (LB) broth, LB agar (Melford), UltraPure Agarose (Thermo Fisher), DMEM with 10% (v/v) foetal bovine serum (Labtech).
- Sterile 10 cm petri dishes and 12-well plates.
- Histology: Paraformaldehyde, ethanol, xylene.
- Histology: Hematoxylin and eosin (H&E) staining kit (Abcam).
- Glycerol stock of microorganism to be investigated (in this example, the *P. aeruginosa* strain PA103).

Required instruments

- Biosafety Cabinet (BSC), Class II.
- Tissue homogenizer (such as a Qiagen Tissue ruptor).
- Gel Imaging System: A Bio-Rad ChemiDoc or equivalent.
- Spectrophotometer: equipped with a 12-well plate reader for absorbance measurements.
- Microtome (histology).

Preparation of bacterial inoculum

2h 25m

1 The day before porcine cornea dissection, inoculate 100 mL of LB broth with a pipette scratch of microorganism to be investigated (*P. aeruginosa* strain PA103 will be discussed in an exemplary protocol) from a glycerol stock (or agar plate, if appropriate). Culture overnight at 37 ºC in a shaker incubator.

10m

2 Take 2 mL of the PA103 overnight culture and add to 20 mL of fresh LB broth. Allow the subculture to expanded in a shaker incubator (37°C) until an OD_{600} of 0.8 is reached.

2h

3 Take 1 mL of subculture and centrifuge for 5 minutes at 5000 **g**. Discard the supernatant and resuspend the bacterial pellet in 10 mL of PBS, yielding a PA103 suspension at a concentration of 10^7 CFU/mL.

Note: When culturing microorganisms other than *P. aeruginosa*, preliminary methods such as serial plating should be performed to accurately quantify their concentrations at various optical densities (OD_{600}).

15m

Preparation of porcine corneas

1h 50m

4 The morning of slaughter, collect porcine eyes from the abattoir and transfer to PBS.

5 Make sure that all regents and equipment used are sterilized (autoclaved where appropriate) before beginning dissections. In a laminar flow hood, use forceps to hold the eye in place and make a small at the edge of the sclera to release ocular pressure and provide an entry point for surgical scissors. Use this procedure for each cornea to be dissected.

5m

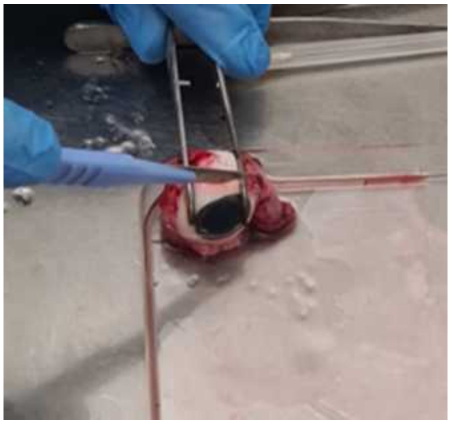

6 Use surgical scissors to cut out the cornea. Maintain 2.5-5 mm of sclera outside the circumference of the cornea.

5m

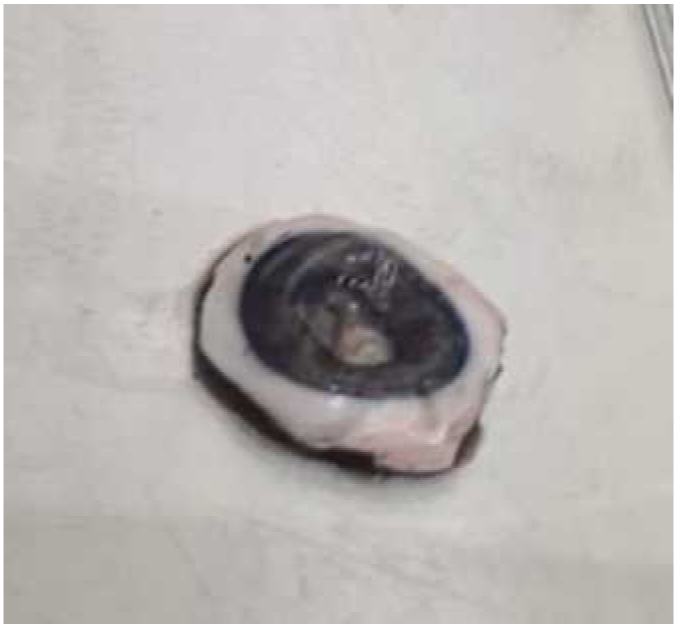

7 From the resulting corneoscleral button, remove the uveal tissue using two sets of forceps. 5m

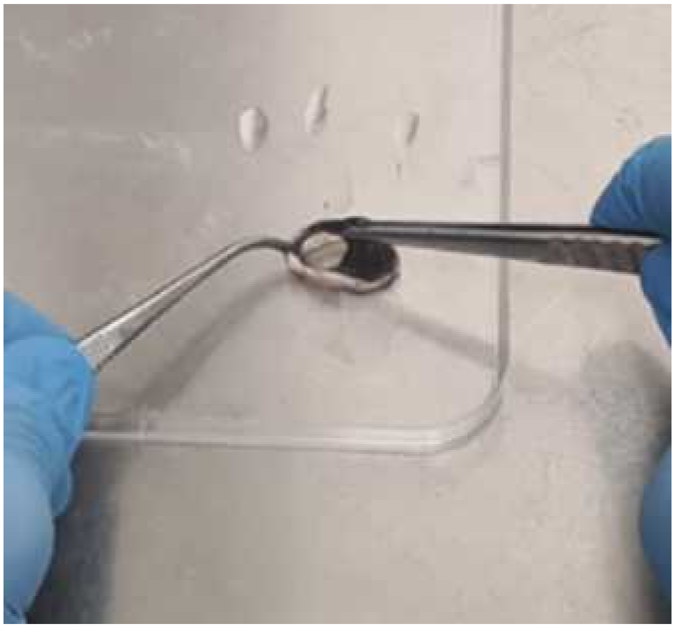

8 For sterilisation, incubate corneas in 10% (v/v) iodinated povidone for 2 minutes. Afterwards, wash the corneas 3 times in PBS. 5m

9 In another 10 cm dish, immerse one side of 10 mm plumbing ring onto dispensed LIQUIBAND topical skin adhesive. Use forceps to compress the edges of the ring to the center of the cornea, ensuring a tight seal. 5m

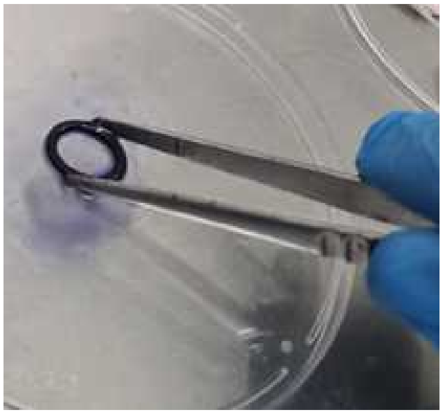

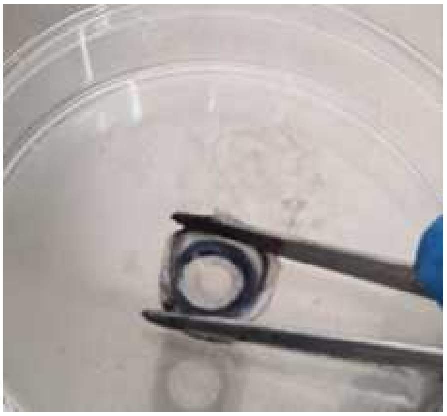

10 Dissolve agarose (0.5 [w/v]) in DMEM using a water bath heated to 65 °C. Allow this solution to cool to approximately 37 °C. 1h

11 Place corneas epithelial side down. Using a pipette, add the cooled agarose DMEN to the endothelial side of corneas to fill the cavity. 10m

12 Once the agarose-DMEN has solidified, transfer the corneas into 12-well plates, epithelial side up. 5m

13 To induce a controlled epithelial wound on the cornea, add 10 µL 70% ethanol (v/v in PBS) to the center of the corneal surface for 10 seconds. Following ethanol exposure, gently scrape ethanol covered epithelium with the edge of a scalpel to simulate mechanical injury. 5m

14 Rince the corneas three times with PBS to ensure complete removal of ethanol and debris. Finally, add 1 mL of DMEM to each well, covering the outer edge of the corneal epithelium without submerging the plumbing ring. 5m

Infection of corneas and topical application of treatment(s) 20m

15 Add 20 µL of the previously prepared bacterial suspension to an Ependorf tube (one Ependorf to be used per cornea). Before addition, ensure that the bacterial suspension is adequately dispersed with a brief vortex. 5m

16 Make up the final volume to 300 µL with PBS, with topical treatments to be investigated also included. 5m

17 Add 150 µL of suspension from step 2 to the centre of each cornea, filling the plumbing ring reservoir. In this example, for *P. aeruginosa* PA103, this equates an inoculum of 1×10^5 colony forming units (CFU) for each cornea. 5m

18 Transfer the corneas, in 12-well plates, to a 37 °C incubator in 5 % CO_2 (v/v). Infection progress should be monitored over the course of several days. Infection progression and extent can vary depending on the species and strain being investigated. It is therefore advised that initial optimization experiments are carried out, inoculating corneas with a range (1×10^2 – 1×10^7) of CFU, observing infection progression at 24 h time points. 5m

Imaging and spectrophotometric analysis of corneal opacity 30m

19 At assay endpoint, take the corneas from the incubator. Using forceps, carefully remove the plumbing rings and rinse the corneas gently in a beaker of PBS. handle the corneas with care, as infection can cause the epithelial layer to become fragile and prone to detachment. Minimize physical contact with the surface to preserve tissue integrity. 10m

20 Image the corneas using a Bio-Rad ChemiDoc (or equivalent), selecting the appropriate gel imaging mode (e.g., "Coomassie Stain" or similar) for capturing qualitative data on ulcer size and density. 10m

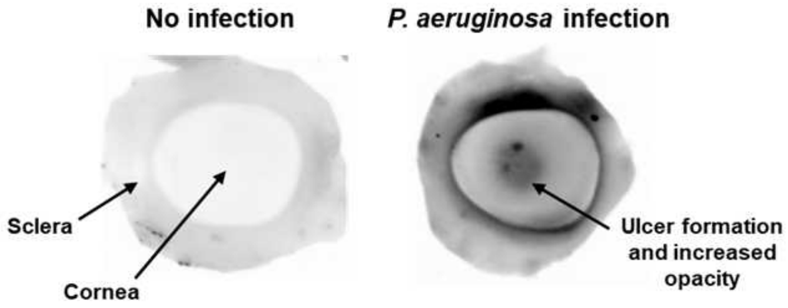

21 To assess ulcer density using a spectrophotometer, place the corneas epithelial side up in 6 or 12-well plates and take optical absorbance readings at 400 nm. 10m

22 For quantitative analysis of ulcer size and density, utilize open-source software such as ImageJ. Use the measurements tool in ImageJ to outline and quantify ulcer areas, applying appropriate thresholding
to assess density and ensure consistent parameters across samples for reliable comparison.

Histological analysis of infected porcine corneas

1d 17h 26m

23 Incubate the corneas in 4% paraformaldehyde (PFA) for 24 h. 1d

24 Dehydrate the corneas through a graded series of ethanol (70%, 90% then 100% [v/v in PBS]). 30m

25 Clear the corneas using xylene and then embedded in paraffin wax. 30m

26 Slice the corneas into 5 µm-thick sections using a microtome, ensuring consistent thickness for reproducibility. 1h

27 Mount the sections onto numbered glass slides, ensuring sequential numbering for reliable comparison between samples. 1h

28 Prepare the samples for staining or labelling by clearing with xylene and hydration through a graded series of ethanol (100%, 95%, 70%, 50% and 0% [v/v in PBS]). 30m

29 For H&E staining: Immerse slides in hematoxylin stain for 5 minutes and then rinse the slides in running tap water for 1-2 minutes to remove excess stain. 10m

30 Differentiate in acid alcohol (1% HCl in 70% ethanol) for a few seconds to remove nonspecific staining then rinse in water. 1m

31 Immerse the slides in 0.1% (v/v) ammonium hydroxide in distilled water for 30 seconds to 1 minute and then rinse with water. 5m

32 Immerse slides in eosin stain for 30 seconds to 2 minutes and then rinse the slides in water to remove the excess stain. 5m

33 Dehydrate the samples by immersion in a graded series of ethanol (70%, 95% and 100% [v/v in PBS] and then clear by incubation in xylene (2 minutes). 30m

34 Add mounting medium to the sample and coverslip.

5m

35 Allow the samples to dry and then view under a light microscope and take images using a camera or slide scanner.

12h

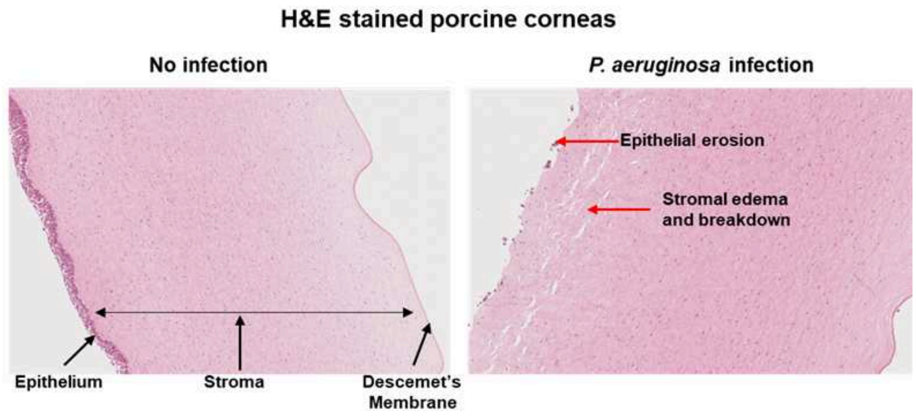

36 ImageJ can be effectively used to quantify epithelial thickness and assess stromal swelling. For epithelial measurements, simple linear measurements can be applied directly on calibrated images. Stromal swelling can be quantified by measuring the white space within the stroma. To achieve this, convert H&E-stained images to 8-bit grayscale and define the stroma as the region of interest (ROI). Set threshold values (e.g., 230–255) to isolate white pixels corresponding to stromal spaces. Calculate the percentage area occupied by white pixels within the ROI to quantify stromal swelling accurately. This approach provides a reproducible and standardized method for image-based corneal analysis.

1h
