# Supplementary figures and images for: Stable topical application of antimicrobials using plumbing rings in an ex vivo porcine corneal infection model
Source: PLoS One. 2025 Apr 3;20(4):e0319911. doi: 10.1371/journal.pone.0319911 (PMC11968109; doi:10.1371/journal.pone.0319911)

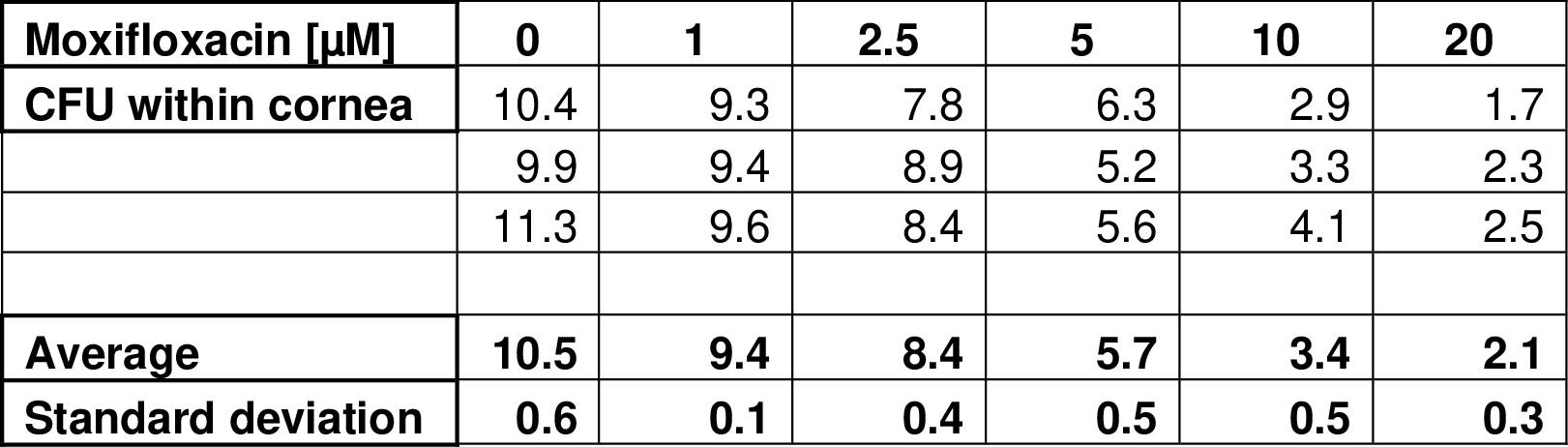

Supplement: S1 Table — PA103 CFU detection in ex vivo porcine corneas after 48h infection and moxifloxacin treatment. Ex vivo porcine corneas were infected with 1 x 10^5^ CFU PA103 for 48 h in the indicated concentrations of moxifloxacin. Whole corneas were homogenized, diluted in PBS followed by plating, overnight incubation at 37 ºC prior to CFU counting. All data collected are presented in the Table along with mean ± SD from 3 independent experiments. (TIF) [file pone.0319911.s002.tif]

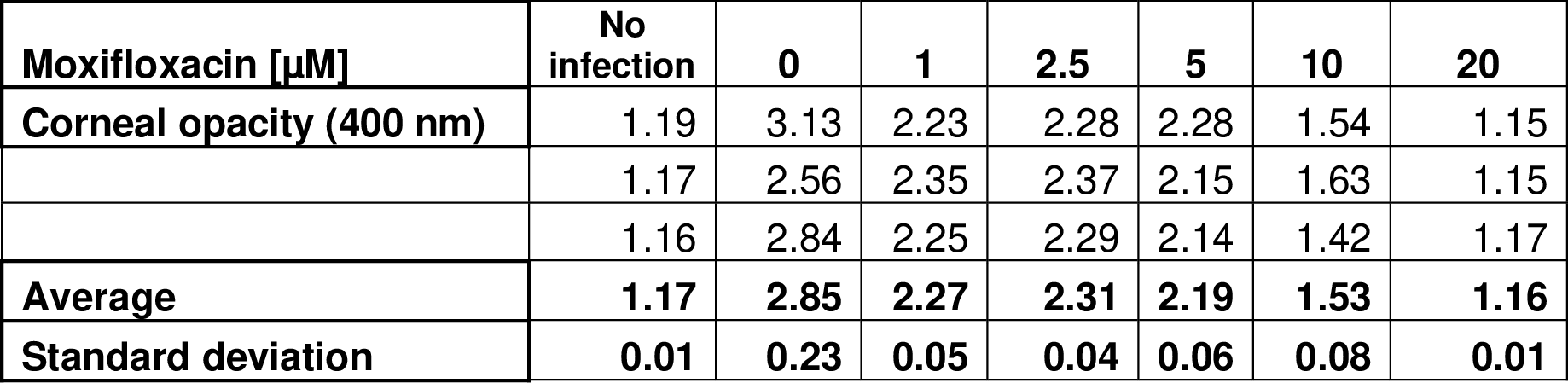

Supplement: S2 Table — Opacity measurement to determine moxifloxacin efficacy in PA103 infected ex vivo porcine corneas. The plumbing rings reservoir on the ex vivo porcine corneas was filled with 150 µ L PBS containing 105 CFU PA103 and varying concentrations of moxifloxacin. After 48h incubation the opacity of the corneas was measured using a spectrophotometer. All data collected are presented in the Table along with mean ± SD from 3 independent experiments. (TIF) [file pone.0319911.s003.tif]

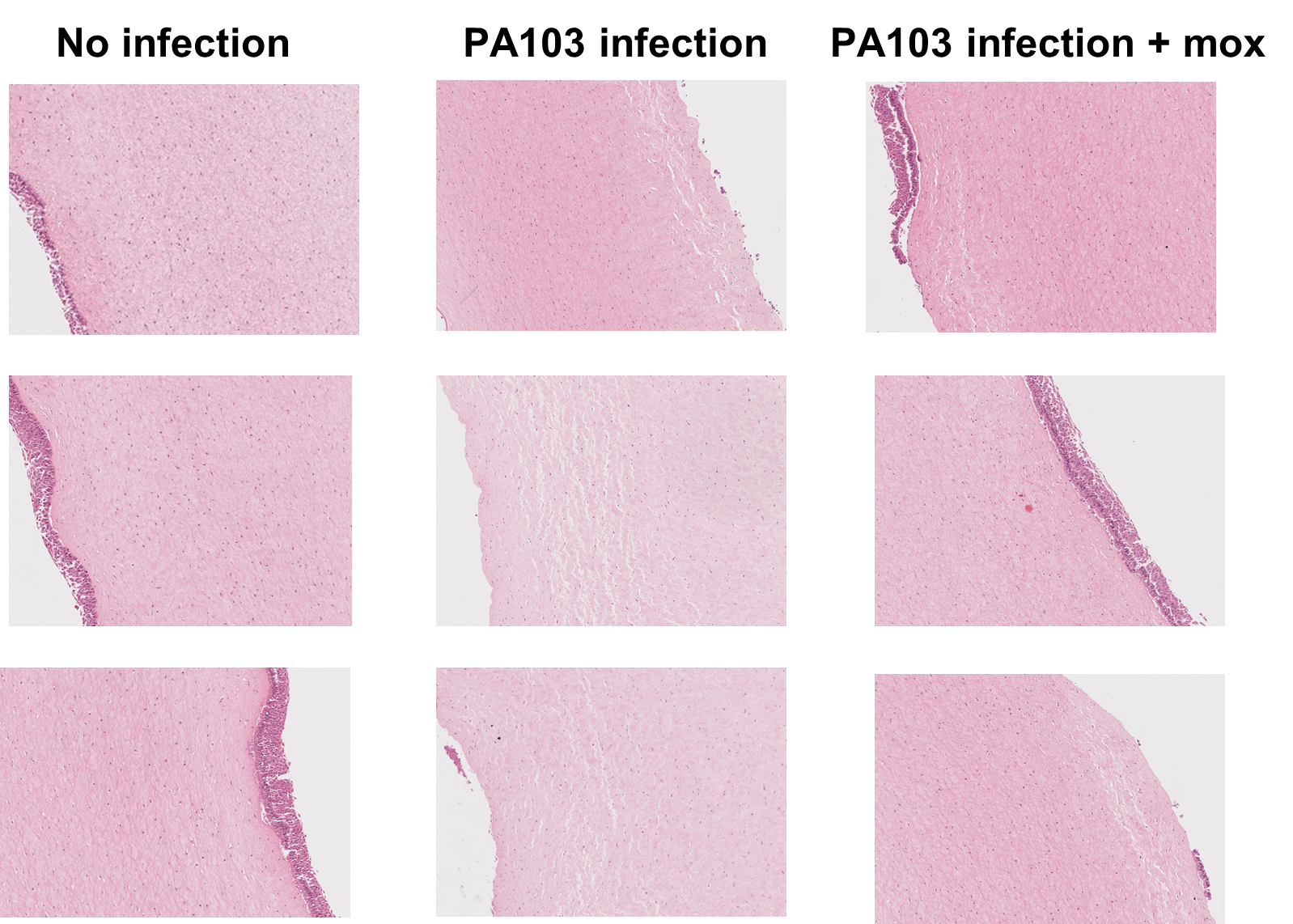

Supplement: S1 Fig — The images used for the quantification of stromal edema from Fig 4B in the main manuscript. Ex vivo porcine corneas were infected with1 x 105 CFU of PA103 for 48h with and without 20 µ M moxifloxacin. Corneas were sectioned, mounted and stained with H&E. A Ventana DP 200 slide scanner was used to capture cross sections of stained corneal sections.) Images of H&E stained corneal sections used for the quantification of stromal swelling (Fig 4B in the main manuscript). Ex vivo porcine corneas were infected with1 x 105 CFU of PA103 for 48h with and without 20 µ M moxifloxacin. Corneas were sectioned, mounted and stained with H&E. A Ventana DP 200 slide scanner was used to capture cross sections of stained corneal sections. (TIF) [file pone.0319911.s004.tif]

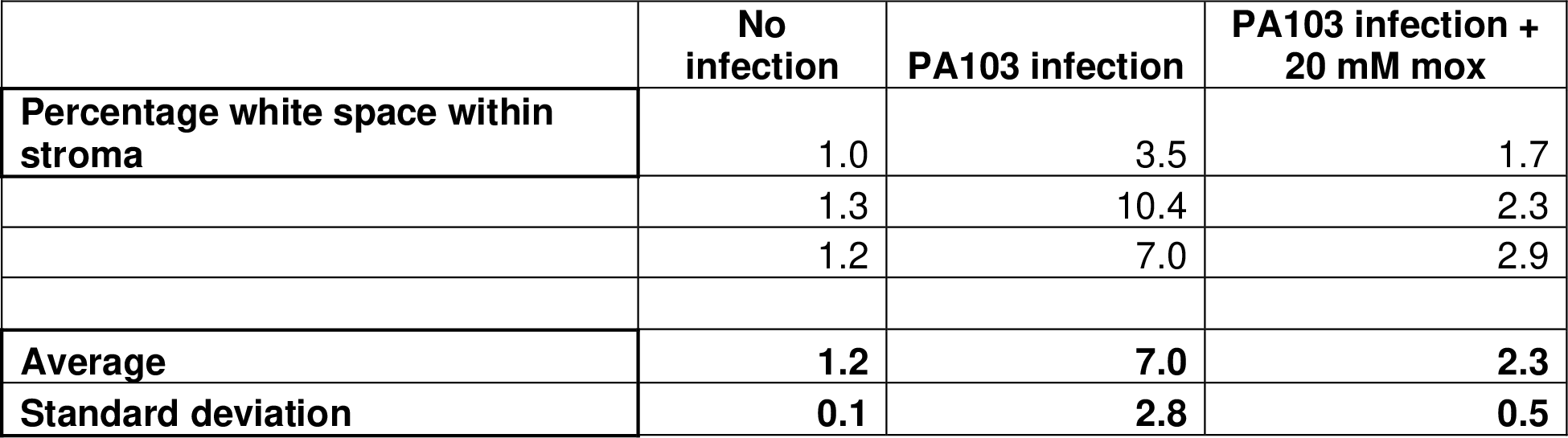

Supplement: S3 Table — The percentage of space within the corneal stroma in response to PA103 infection was assessed using ImageJ. The stroma of the corneal sections was designated as the region of interest (ROI) and converted to 8-bit grayscale for analysis. A threshold range of 230–255 was applied to identify and quantify white space within the stroma. The resulting data are presented in the table, which is derived from the 3 images for each condition shown in S1 Fig. (TIF) [file pone.0319911.s005.tif]
