# Supplementary material for: Stable topical application of antimicrobials using plumbing rings in an ex vivo porcine corneal infection model
Source: PLoS One. 2025 Apr 3;20(4):e0319911. doi: 10.1371/journal.pone.0319911 (PMC11968109; doi:10.1371/journal.pone.0319911)
Supplement: Attachment — Submitted filename: Comments.pdf [file pone.0319911.s006.pdf]

This manuscript describes an improved protocol for studying microbial keratitis (MK) using an ex vivo porcine corneal infection model. The authors adapted an existing protocol by incorporating plumbing rings to maintain localized application of pathogens and therapeutic agents on the corneal surface. The study focuses on *Pseudomonas aeruginosa* infection and evaluates the efficacy of moxifloxacin in reducing bacterial load, corneal opacity, and tissue damage. The findings demonstrate the practicality and reliability of this model for testing antimicrobial treatments, emphasizing its potential applications in ocular research.

Major point:

- The introduction effectively highlights the significance of microbial keratitis and the advantages of ex vivo models. However, the rationale for selecting plumbing rings over other methods (e.g., glass molds) could be elaborated.
- Consider briefly addressing the clinical relevance of the findings, such as potential applications for human ocular infections.
- The CFU analysis in Figure 2 provides compelling evidence of moxifloxacin's efficacy. Could the authors clarify whether the dose-dependent reduction was statistically significant across all tested concentrations?
- Figure 3 shows the restoration of corneal clarity at 20 μM moxifloxacin. The discussion notes incomplete bacterial clearance despite reduced opacity. Can the authors speculate further on the threshold of bacterial load required to induce opacity?
- The histological analysis (Figure 4) effectively demonstrates structural preservation with moxifloxacin treatment. However, additional quantitative metrics, such as epithelial thickness or stromal edema, could strengthen the findings.
- The preparation of bacterial inoculum and porcine corneas is described in detail. However, the use of 70% ethanol for epithelial debridement might alter corneal properties. Have the authors considered alternative methods?
- The choice of 10 mm plumbing rings is practical, but information on how ring size or adhesive type affects results would be beneficial.
- The methods lack specific information about the statistical analysis software and parameters. Please provide these details.
- The discussion addresses key limitations, including the absence of immune responses in ex vivo models. Could the authors propose strategies for overcoming these limitations, such as co-culture systems with immune components?
- The statement about reduced PcrV expression with moxifloxacin is intriguing but not directly tested in this study. Consider elaborating on how this mechanism aligns with the observed outcomes.

- The discussion could benefit from a stronger conclusion regarding the translational potential of the findings for human therapy.

Minor opinions:

- Abstract: Line 12 “interaction more accurately than” – consider rephrasing for clarity.
- Introduction: Line 43, “*P. aeruginosa* possesses an array of virulence factors” – consider specifying which are most relevant to MK.
- Methods: Line 127, “a concentration of 10^7 CFU/mL” – use superscripts for scientific notation.
- Figures: Ensure figure legends are self-explanatory without requiring text from the main body for context.
- Formatting: Verify consistent use of italicization for species names throughout the manuscript.
