# Supplementary material for: Stable topical application of antimicrobials using plumbing rings in an ex vivo porcine corneal infection model
Source: PLoS One. 2025 Apr 3;20(4):e0319911. doi: 10.1371/journal.pone.0319911 (PMC11968109; doi:10.1371/journal.pone.0319911)
Supplement: Attachment — Submitted filename: Response to Reviewers.docx [file pone.0319911.s007.docx]

Dear Editor,

*Please find within this letter all our responses along with details of the revisions to the manuscript. We first address the editors’ issues, which we hope can now be resolved, followed by our responses to the reviewer comments. We extend our sincere thanks to the editor for their diligence and for their feedback in the improvement of our manuscript.*

*As requested, you will find a marked-up copy of the revised manuscript, as well as a final copy without tracked changes. We have since submitted our protocol to protocols.io, as recommended, and have reserved a DOI (DOI: dx.doi.org/10.17504/protocols.io.eq2ly6nkmgx9/v1).*

*All data for this study can be found both within the manuscript and the additional supplementary appendix files we have now provided.*

1. Please ensure that your manuscript meets PLOS ONE's style requirements, including those for file naming. The PLOS ONE style templates can be found at
   https://journals.plos.org/plosone/s/file?id=wjVg/PLOSOne_formatting_sample_main_body.pdf and 
   <https://journals.plos.org/plosone/s/file?id=ba62/PLOSOne_formatting_sample_title_authors_affiliations.pdf>

*We have checked the style requirements and hope that it is now fitting.*

2. Thank you for stating the following financial disclosure:
   Funded by The Fight for Sight Carmen Butler-Charteris Charitable Trust. Project: 5175/5176. Please state what role the funders took in the study.  If the funders had no role, please state: ""The funders had no role in study design, data collection and analysis, decision to publish, or preparation of the manuscript."" 
   If this statement is not correct you must amend it as needed. 
   Please include this amended Role of Funder statement in your cover letter; we will change the online submission form on your behalf.

*The statement "The funders had no role in study design, data collection and analysis, decision to publish, or preparation of the manuscript" is correct. The authors would be most grateful if this could be changed in the online submission form.*

3. We note that you have indicated that there are restrictions to data sharing for this study. PLOS only allows data to be available upon request if there are legal or ethical restrictions on sharing data publicly. For more information on unacceptable data access restrictions, please see <http://journals.plos.org/plosone/s/data-availability#loc-unacceptable-data-access-restrictions>.

*There are no restrictions on data sharing for this study. All data is now included in the manuscript and supplementary information. On behalf of the authors, I apologise for this oversight.*

4. In the online submission form, you indicated that The data underlying the results presented in the study are available on request from either Daniel Foulkes (daniel.foulkes@liverpool.ac.uk) or Stephen Kaye ([sbkaye@liverpool.ac.uk](mailto:sbkaye@liverpool.ac.uk)). All PLOS journals now require all data underlying the findings described in their manuscript to be freely available to other researchers, either 1. In a public repository, 2. Within the manuscript itself, or 3. Uploaded as supplementary information.
   This policy applies to all data except where public deposition would breach compliance with the protocol approved by your research ethics board. If your data cannot be made publicly available for ethical or legal reasons (e.g., public availability would compromise patient privacy), please explain your reasons on resubmission and your exemption request will be escalated for approval.

*All of the study data can now be found in manuscript and new supplementary information files (S2 and S3).*

5. Please include captions for your Supporting Information files at the end of your manuscript, and update any in-text citations to match accordingly. Please see our Supporting Information guidelines for more information: <http://journals.plos.org/plosone/s/supporting-information>.

*Captions for all supporting information files have now been included at the end of the manuscript.*

**Responses to Reviewer 1**

*We sincerely thank the reviewer for their thoughtful comments and valuable feedback. We hope that we have addressed all comments satisfactorily. Should the reviewer have further suggestions or areas they believe warrant elaboration, we would be happy to address them.*

Major points:

1. The introduction effectively highlights the significance of microbial keratitis and the advantages of ex vivo models. However, the rationale for selecting plumbing rings over other methods (e.g., glass molds) could be elaborated.

*The authors thank the reviewer for this observation and agree that further highlighting the advantages of using plumbing rings over glass moulds outlines them as a strong alternative. To make the logistical limitations more clear for the use of glass moulds (requiring a skilled glass blower and specialised facilities), we have introduced a separate paragraph. We have also elaborated on the advantages of using plumbing rings and medical adhesive – “The application of plumbing rings with medical adhesive to create an apical reservoir on ex vivo corneas does not require extensive technical skill. Moreover, the reagents used are inexpensive and readily available to any lab.”*

2. Consider briefly addressing the clinical relevance of the findings, such as potential applications for human ocular infections.

*We have now addressed this and added appropriate text to the abstract, introduction and discussion sections.*

3. The CFU analysis in Figure 2 provides compelling evidence of moxifloxacin’s efficacy. Could the authors clarify whether the dose-dependent reduction was statistically significant across all tested concentrations?

*Statistical analysis was indeed performed and the reduction in CFU was determined to be statistically significant. We have aligned the asterisks above the data in Figure 2 to improve clarity.*

4. Figure 3 shows the restoration of corneal clarity at 20 µM moxifloxacin. The discussion notes incomplete bacterial clearance despite reduced opacity. Can the authors speculate further on the threshold of bacterial load required to induce opacity?

*This is a good and interesting question, albeit difficult to answer.* *Extent of bacterial load may not directly translate to a proportional increase in opacity. We hypothesise that the extent of opacity stems from the virulence of the bacteria and cannot necessarily be pinpointed to a measure of growth. The bacterial virulence factors modulate the structure of the epithelium and endothelium resulting in loss of translucent properties. In this regard, we hypothesise that high numbers of bacterial CFU with lower pathogenicity would not cause as much opacity as lower bacterial CFU with increased virulence output. We have now included this observation within the results and discussion – “The extent of bacterial load may not directly translate to a proportional increase in corneal opacity. We hypothesise that the degree of opacity is primarily driven by bacterial virulence factors rather than the bacterial load itself. These factors modulate the structure of the epithelium and endothelium, leading to a loss of corneal transparency. As such, high CFU counts with low virulence output may result in less opacity than lower CFU counts with higher virulence.”*

5. The histological analysis (Figure 4) effectively demonstrates structural preservation with moxifloxacin treatment. However, additional quantitative metrics, such as epithelial thickness or stromal edema, could strengthen the findings.

*We thank the reviewer for their valuable feedback. While the preservation of the epithelium is visually clear, the extent of stromal swelling is less apparent and difficult to assess by eye. To address this, we have incorporated additional quantitative analysis into our study. In the revised manuscript, we used ImageJ to quantify stromal swelling in corneal sections by measuring the percentage of white space within the stroma. The percentage area of white pixels in the stromal region was calculated and subsequently analysed using GraphPad Prism. These results are now presented in Figure 4B.*

6. The preparation of bacterial inoculum and porcine corneas is described in detail. However, the use of 70% ethanol for epithelial debridement might alter corneal properties. Have the authors considered alternative methods?

*In previous studies, we have employed both the needle scratch and 70% ethanol debridement methods. Whilst the needle scratch method is effective, we also find it to be more technically challenging and thus less reproducible. We highlight in this study that both methods can be used but advocate the latter for consistency. With this, to the manuscript we have added “In this study, we employed ethanol debridement as we found this to be less technically challenging than needle scratching and more reproducible.” As for the question of the potential alteration of corneal properties, we observed by histological analysis that the ethanol debridement method, after incubation, did not result in any obvious morphological changes.*

7. The choice of 10 mm plumbing rings is practical, but information on how ring size or adhesive type affects results would be beneficial.

*We thank the reviewer for their comment as this is an interesting point to raise, which we can address and improve the quality of the publication. The size of 10 mm is important as it provides a substantial area for inoculum and topical treatments so that analysis outputs can be maximised. 10 mm plumbing rings were consistently able to be fitted to all porcine corneas tested, whereas larger sizes would sometimes become imposed onto the sclera, due to variations in eye sizes obtained from the abattoir. Indeed, smaller size plumbing rings may be used if a smaller initial area of infection is desired, such as for studying infection with herpes. This has now been discussed in the manuscript.*

8. The methods lack specific information about the statistical analysis software and parameters. Please provide these details.

*Although statistical analysis information is mentioned in the figure legends, we have now provided a section outlining the statistical analysis in the methods (section 2.8).*

9. The discussion addresses key limitations, including the absence of immune responses in ex vivo models. Could the authors propose strategies for overcoming these limitations, such as co-culture systems with immune components?

*An additional paragraph has been included in section 4.2, where we have discussed co-culture systems, the potential for neutrophil transepithelial migration assays and immune cells, cytokines, and antimicrobial factors using microfluidic platforms.*

10. The statement about reduced PcrV expression with moxifloxacin is intriguing but not directly tested in this study. Consider elaborating on how this mechanism aligns with the observed outcomes.

*We have expanded results and discussion section 3.2.*

11. The discussion could benefit from a stronger conclusion regarding the translational potential of the findings for human therapy.

*The conclusion now outlines the clinical translation potential of the developed model. “With this, we present a highly accessible and improved ex vivo infection model, which may lead to* *better clinical translation of prospective treatments to reduce the burden of MK”.*

**Minor comments:**

1. Abstract: Line 12 “interaction more accurately than” – consider rephrasing for clarity.

*The sentence has been re-written for improved clarity.*

2. Introduction: Line 43, “P. aeruginosa possesses an array of virulence factors” – consider specifying which are most relevant to MK.

*The paragraph has been revised to discuss the roles of Type III secretion system (T3SS) effectors, with a particular focus on ExoU, whose expression is strongly correlated with increased disease severity and poorer clinical outcomes. Additionally, the roles of ExoS and secreted proteases (LasB, protease IV, and PASP) are highlighted as key drivers of MK pathogenesis.*

3. Methods: Line 127, “a concentration of 107 CFU/mL” – use superscripts for scientific notation.

*This has now been amended.*

4. Figures: Ensure figure legends are self-explanatory without requiring text from the main body for context.

*The Figure legends have been revised.*

5. Formatting: Verify consistent use of italicization for species names throughout the manuscript.

*The manuscript has been fully checked for proper italicization.*

**Responses to Reviewer 2**

*We thank the reviewer for their encouraging comments on our manuscript. We are pleased that the reviewer recognises the significance of our work in addressing the global challenge of MK and the urgent need for improved models to study this disease. We are also grateful for the acknowledgment of the advantages offered by our ex vivo porcine corneal model, including its simplicity, cost-effectiveness, and ethical acceptability.*

*We share the reviewer’s perspective on the limitations of the porcine corneal model and have strived to provide a balanced discussion of its strengths and constraints. Once again, we thank the reviewer for their positive feedback.*

*Yours sincerely,
Daniel Morgan Foulkes Department of Eye and Vision Science, Institute of Life Course and Medical Sciences, University of Liverpool, U.K.*
